# Do health service contacts with community health workers influence the intention to use modern contraceptives among non-users in rural communities? Findings from a cross-sectional study in Nigeria

**DOI:** 10.1186/s12913-023-09032-3

**Published:** 2023-01-10

**Authors:** Bola Lukman Solanke, Olufemi O. Oyediran, Abayomi Folorunso Awoleye, Oluwayemisi Elizabeth Olagunju

**Affiliations:** 1 Department of Demography and Social Statistics, Obafemi Awolowo University, Ile-Ife, Nigeria; 2 Department of Nursing Science, Obafemi Awolowo University, Ile-Ife, Nigeria

**Keywords:** Community health workers, health service contacts, intention to use contraceptives, rural women, Nigeria

## Abstract

**Background:**

Studies in many developing countries have shown that community health workers (CHWs) are valuable for boosting contraceptive knowledge and usage. However, in spite of the evidence, studies in Nigeria have rarely examined whether in the absence of skilled health personnel such as doctors and nurses in rural and remote communities, the health service contacts of non-users with CHWs drive the intention to use modern contraceptives. This study, therefore, examines the extent to which health service contacts with CHWs are associated with the intention to use modern contraceptives among non-users in rural communities of Nigeria.

**Methods:**

This study adopted a descriptive cross-sectional design. Data were extracted from the most recent Nigeria Demographic and Health Survey (NDHS). The study analyzed a weighted sample of 12,140 rural women. The outcome variable was the intention to use modern contraceptives. The main explanatory variable was health service contacts with CHWs. Statistical analyses were performed at three levels with the aid of Stata version 14. Three multivariable regression models were estimated using an adjusted Odds Ratio (aOR) with a 95% confidence interval. Statistical significance was set at *p* < 0.05.

**Results:**

Findings showed that more than a quarter (29.0%) of women intends to use modern contraceptives. Less than one-fifth (15.9%) of the women had health service contacts with CHWs. In Model 1, women who had health service contacts with CHWs were more likely to intend to use modern contraceptives (aOR =1.430, 95% CI: 1.212–1.687). Likewise, in Model 2, women who had health service contacts with CHWs had a higher likelihood of intending to use modern contraceptives (aOR = 1.358, 95% CI: 1.153–1.599). In Model 3, the odds of intention to use modern contraceptives were higher among women who had health service contacts with CHWs (aOR =1.454, 95% CI: 1.240–1.706).

**Conclusion:**

In rural areas of Nigeria, health service contacts with CHWs are significantly associated with the intention to use modern contraceptives. Family planning programmers should leverage the patronage of CHWs for the purpose of family planning demand generation in rural areas.

## Background

The intention to use a modern contraceptive indicates the proportion of all women of reproductive age who are not currently using a modern method but have the intent to become modern contraceptive users in the future. This indicator is crucial to family planning programming particularly the family planning demand generation in Nigeria for two reasons. One, it provides information about the current level of non-use of modern contraceptives in the country. Evidence suggests that the non-use of modern contraceptives is not only high in Nigeria but also higher in rural areas [1], as well as in the northern parts of the country [2] compared to the urban and southern parts of the country. Two, it provides information about the extent to which current non-users plan to utilize modern contraceptives in the future. Such information drives the expansion of existing family planning service delivery to levels that anticipates and accommodate future demand for modern contraceptives. It also justifies the need for continued research focusing on the intention to use modern contraceptives among childbearing women.

In Nigeria, modern contraceptive prevalence remained low among young and older women of reproductive age [3, 4] and young and older men [5] though with substantial state and regional variations across the country [6, 7]. For instance, while modern contraceptive use was 12% among currently married women, the use was 28% among unmarried women [7]. Also, the prevalence rate was lower than 10% in northern Nigeria compared to more than 15% in southern Nigeria [2, 3, 8]. Furthermore, modern contraceptive use was 26% among currently married women in urban areas compared to 10% among a similar group of women in rural areas [7]. Evidence also shows that a high level of unmet need for contraception exists in the country [8, 9]. For instance, a recent study found that the unmet need for contraception exceeded 20% in many states of Nigeria. The 2018 Nigeria Demographic and Health Survey reported the unmet need for contraception to be 35% in Cross River State, 33% in Edo State, and 10% each in Anambra and Adamawa states [7].

These pose a significant threat to achieving the national contraceptive prevalence target as declared in the current national policy on population for sustainable development [10]. The policy seeks to increase the modern contraceptive prevalence rate to 27% by 2020, and successively gain an additional 2% increase yearly until 2030. The policy was built on the 2014 national family planning blueprint, which revised the national contraceptive prevalence goal from 36 to 27% by 2024 [11, 12]. The development of the national family planning blueprint was driven with a view to achieving these targets through improve family planning demand generation and service delivery, particularly in the rural areas of the country where the use of modern contraceptives is lower compared to the urban areas.

The family planning blueprint included sets of workable activities intended to generate more demand for family planning. This includes the development of the national family planning communication plan, the rejuvenation of the national health promotion forum, and the launching of the new family planning logo codenamed ‘the Green Dot’. These activities utilized several health and non-health resources such as health communication experts, media organizations, the national orientation agency, faith-based organizations, and existing health agencies. These activities have improved public awareness of family planning in the country [12]. Nevertheless, some health resources that could further enhance the adoption of modern contraceptives by current non-users are still underutilized in the existing framework. One such health resource is the pool of Community Health Workers (CHWs). This refers to individuals who live and work in rural and remote communities to provide basic health services [13, 14]. In Nigeria, there are four cadres of CHWs, namely, community health officers, community health extension workers, junior community health extension workers, and community resource persons [15]. The CHWs received limited health-related training certified by the Community Health Practitioners Registration Board of Nigeria. This enables CHWs to provide recognized specific primary health care both in a health facility and as well as in the community [16, 17], to support the services provided by skilled health personnel such as doctors and nurses/midwives, who in many instances are not readily available in rural and remote communities [18–20]. The range of duties performed by CHWs includes but is not limited to supporting maternal and child health [21–24], helping to control communicable and non-communicable diseases [25], treatment of endemic diseases [26], promoting adequate nutrition [27], providing support during outbreaks of pandemics [17, 28], home visits, referral of patients, and disease surveillance [29].

More importantly, studies in many developing countries have shown that CHWs are valuable for boosting contraceptive usage in rural communities [30–32]. In Nigeria, a recent study [33] based on the PMA2020 survey in six states investigated the positive impact of CHWs on modern contraceptive use among childbearing women in rural parts of the country. The study revealed that women visited by CHWs reported higher usage of modern contraceptives which corroborates an earlier finding in India that health workers’ outreach may influence intention to use contraceptives [34]. In recognition of the value of CHWs in boosting contraceptive prevalence in the country, the Nigeria National Council on Health [NCH] (2012) approved that community health extension workers be allowed to provide injectable contraceptives in communities, which is a practice already found to be impactful in other climes [35, 36].

In spite of the evidence of CHWs relevance to contraceptive usage in the country, studies have rarely examined whether in the absence of skilled health personnel such as doctors and nurses in rural and remote communities, the health service contacts of non-users with CHWs drive the intention to use contraceptives. This is important because, in many rural and remote communities of Nigeria, skilled health personnel is not readily available at health facilities in addition to other sundry challenges such as long distances to the facility, lack of essential drugs, and poverty [18, 20]. These challenges discourage the use of essential healthcare services in rural areas. The challenges are now aggravated by widespread insecurity of lives and properties in the country [37, 38] which has led to increasing non-availability of skilled health personnel in rural areas of the country. Some rural dwellers may thus be limited to contacting CHWs for antenatal, delivery, postnatal checks, family planning, and treatment of diverse ailments. Often the health service contacts take place in non-clinical spaces, and sometimes the CHWs visit homes, markets, and farmlands to provide health education and basic services to the people. Hence, if CHWs have sufficient training and education about modern contraceptives, the health service contacts with non-users could be an avenue to talk about family planning, clear misconceptions about family planning, and addresses other family planning concerns of the rural populace. The study, therefore, examines the extent to which health service contacts with CHWs are associated with the intention to use modern contraceptives among non-users in rural communities. Findings will provide further inputs for strengthening the family planning demand generation activities of the family planning blueprint. It will also shed light on how CHWs could contribute to the achievement of the national contraceptive targets sets for 2030 and 2050 in Nigeria.

## Methods

### Design and Data

This study adopted a descriptive cross-sectional design using a quantitative approach to determine whether the health service contacts of non-users with CHWS are associated with the intention to use contraceptives. The women’s datasets analyzed in the study were extracted from the 2018 Nigeria Demographic and Health Survey (NDHS). The 2018 NDHS is the current (seventh) round of the Demographic and Health Survey (DHS) program implemented in Nigeria by the National Population Commission (NPC) with the collaboration of related national agencies, and the technical support of the Inner-City Fund (ICF) obtained through the DHS Program **[7]**. The DHSs are conducted across developing countries to build national capacity for the production of demographic and health data, and to provide estimates of demographic and health characteristics required for the monitoring of the Sustainable Development Goals in the participating countries [39, 40]. The 2018 NDHS thus provides internationally comparable demographic and health information that remains valid until the next round of the DHS in 2023. Comprehensive information about the methodology of the 2018 NDHS has been published and made available in the public domain via https://dhsprogram.com/pubs/pdf/FR359/FR359.pdf.

### Population and Sample

The 2018 NDHS covered 41,821 women of childbearing age. However, some of the women who were not relevant to the current study were excluded. Women excluded include all urban women (16,984), women currently using a method of contraception (8300), infecund women (572), and women who had health service contacts with skilled health personnel such as doctors and nurses/midwives (3121). Thus, the study analyzed a weighted sample of 12,140 women. This represents all women in the 2018 NDHS who were rural dwellers, reported CHWs as the provider of antenatal care, delivery, or postnatal care, and were not currently using a method of contraception. The sample was weighted using the weighting factors available in the dataset.

### Research Variables

The outcome variable in the study was the intention to use modern contraceptives. This was derived from responses to the question: “Do you think you will use a contraceptive method to delay or avoid pregnancy at any time in the future?” The responses were in three categories, namely, intend to use later, unsure about future use, and do not intend to use modern contraceptives later. The study focused on women who were sure of their intention to use modern contraceptives later. We, therefore re-grouped the outcome variable into two categories, namely, ‘intend to use’ or ‘otherwise’. This measure was consistent with how the intention to use contraceptives was operationalized in existing studies [41–44]. The main explanatory variable in the study was health service contacts. This was measured among childbearing women who received antenatal care, delivery care, or postnatal care solely from any of the cadres of CHWs and not from skilled health personnel. The variable was grouped into two categories of ‘health service contact’ or ‘no health service contact’ Three other sets of explanatory variables were included in the analysis for the purpose of making the study findings robust.

One, eight individual demographic and social characteristics were examined. These are maternal age group (15–24, 25–34, and 35 years or older), timing of marriage (early - before 18 years or not early – 18 years or older), maternal education (none, primary, secondary, and higher), parity (primiparity [one child], multiparity [two to four children], and grand multiparity [five or more children]), working status (employed or unemployed), fertility desire (wants within 2 years, wants after 2 years, wants but unsure of timing, undecided, and wants no more) and, mass media exposure (low, moderate, and high). This was derived from the frequency of reading newspapers, listening to the radio, or watching television. A total of nine points was generated and subsequently divided into three equal parts to indicate low, moderate, and high exposure. Also, religion (Christianity, Islam, and others) was examined. Previous studies [42, 43, 45–47] have shown that these variables are important correlates of the intention to use contraceptives.

Two, six household characteristics were examined. These are household wealth quintile (poorest, poorer, middle, richer, and richest), partners’ education (none, primary, secondary, and higher), financial autonomy (autonomous or not autonomous), and healthcare autonomy (autonomous or not autonomous). Healthcare and financial autonomy were derived from responses to who had the final say on women’s own health and spending of the respondents’ cash earnings. Women who solely had the final say or jointly with partners were grouped as ‘autonomous’ and ‘not autonomous’ if otherwise. Safer sex negotiation (able to negotiate or unable to negotiate) was also included. Women who reported they could refuse sex from their partners or could ask partners to use a condom during intercourse were categorized as ‘able to negotiate’. The last household factor included was the decision-maker for the non-use of contraceptives (respondent, partner, joint, and others). These variables are included to reveal the power dynamics within the household in patriarchal societies such as Nigeria, which have been established in existing studies as important influencing factors on women’s use or non-use of modern contraceptives [48–51].

Three, some variables were selected for statistical control in the study. These are attitudes to wife-beating (not acceptable if the respondent did not justify wife-beating in any circumstance or acceptable if the respondent justifies wife-beating in specific circumstances), visitation by a family planning worker in the last 12 months (visited or not visited), pregnancy termination experience (never or ever experienced), and geo-political zone of residence. These variables have been linked to the intention to use modern contraceptives in previous studies [41, 42]. Pregnancy termination experience was included because evidence suggests that women who have experienced induced abortion, may develop an interest in the use of contraceptives especially when such women have been exposed to postabortion care and counselling [52, 53].

### Data Analysis

Statistical analyses were performed at three levels with the aid of Stata version 14 [54]. At the first level, descriptive statistics were used to present sample characteristics and the intention to use modern contraceptives. At the second level, the explanatory variables were cross-tabulated with the outcome variable to assess variations in the intention to use contraceptives due to variations in the explanatory variables. Two bivariate analyses were further performed to select variables for inclusion in the final stage of data analysis. Firstly, a binary logistic regression using the unadjusted Odds Ratio (uOR) with a 95% confidence interval was used to assess associations between the explanatory and outcome variables. The control variables were excluded from the assessment. Only variables that reveal significance at *p* < 0.025 were selected.

Two, a Variance Inflation Factor (VIF) was performed to check the extent of multicollinearity among the variables. Variables were selected into the multivariable model on the basis of either showing significance by the estimates of the uOR or having a VIF score of less than ten. This was done to maintain the statistical principle that a variable with a VIF score of 10 or higher scores signifies the presence of multicollinearity [55]. At the third stage of data analysis, three multivariable regression models were estimated using an adjusted Odds Ratio (aOR) with a 95% confidence interval. Three models were estimated for the purpose of assessing the strength of health service contacts given the introduction of other variables into the successive models. Model 1 controlled for the individual demographic and social characteristics, while Model 2 controlled for the individual characteristics, and household characteristics. Model 3 was the full model which included all the research variables. Statistical significance was set at *p* < 0.05.

## Results

### Univariate Results

Table 1 presents the demographic and social profile of the respondents. More than a quarter (29.0%) of the respondents intends to use modern contraceptives later. Less than one-fifth of them (15.9%) had health service contact with CHWs, while the majority had no health service contact with CHWs. The proportions of women in older age groups were higher compared to the proportion in the younger age group of 15–24 years. The majority (71.6%) of the respondents reported early marriage while more than a quarter (28.4%) of the women reported otherwise. The proportion of the respondents with a desire for an additional child was high though those who desired to have another child within 2 years were slightly more than those who desired another child after 2 years. The majority of the women (63.3%) had no formal education. Also, the majority (65.3%) were employed at the time of the survey. More than half of the respondents (55.5%) had low mass media exposure. Likewise, more than one-third of them (37.7%) had moderate mass media exposure. Muslim women were dominant among the respondents. Nearly half (46.5%) of the respondents were grand multiparous.

**Table 1:** Demographic and social profile of respondents

Characteristic	Number of women	Percent	Characteristic	Number of women	Percent
**Intention to use contraceptives**			**Household wealth**		
Intends to use	3520	29.0	Poorest	4491	37.0
Otherwise	8620	71.0	Poorer	3989	32.9
**Health service contacts**			Middle	2240	18.4
No contact with CHW	10,206	84.1	Richer	1015	8.4
Contact with CHW	1934	15.9	Richest	406	3.3
**Maternal age group**			**Partners’ education**		
15–24 years	2820	23.2	None	7559	62.3
25–34 years	4568	37.6	Primary	1662	13.3
35 +	4752	39.2	Secondary	2324	19.1
**Timing of first marriage**			Higher	646	5.3
Early	8694	71.6	**Healthcare autonomy**		
Not early	3446	28.4	Not autonomous	8945	73.7
**Parity**			Autonomous	3195	26.3
Primiparity	3405	28.1	**Safer sex negotiation**		
Multiparity	3083	25.4	Unable to negotiate	7029	57.9
Grand multiparity	5652	46.5	Able to negotiate	5111	42.1
**Fertility desire**			**Decision-maker for non-use of contraceptives**		
Wants within 2 years	4217	34.7	Woman only	3659	30.1
Wants after 2 years	3667	30.2	Partner only	2242	18.5
Wants unsure of timing	560	4.6	Joint	3491	28.8
Undecided	815	6.7	Others	2748	22.6
Want no more	2881	23.7	**Visitation by family planning worker**		
**Maternal education**			Not visited	10,375	85.5
None	8301	63.3	Visited	1765	14.5
Primary	1806	14.9	**Attitude to wife-beating**		
Secondary	1806	14.9	Not acceptable	6831	56.3
Higher	227	1.9	Acceptable	5309	43.9
**Working status**			**Pregnancy termination**		
Unemployed	4218	34.7	Never experienced	10,484	86.4
Employed	7922	65.3	Ever experienced	1656	13.6
**Mass media exposure**			**Geo-political zone of residence**		
Low	6733	55.5	North-central	1778	14.6
Moderate	4582	37.7	North-east	2801	23.1
High	825	6.8	North-west	5552	45.7
**Religion**			South-east	407	3.4
Christianity	3292	27.1	South-south	1170	9.6
Islam	8750	72.1	South-west	432	3.6
Others	98	0.8			
**Financial autonomy**					
Not autonomous	6638	54.7			
**Autonomous**	5502	45.3			
**Total**	**12,140**	**100.0**			

Source: Authors’ analysis based on Nigeria Demographic and Health Survey

Most of the women belong to either the poorest or poorer household wealth groups. The majority (62.3%) of respondents’ partners were not formally educated. While the majority (73.7%) of the respondents had no healthcare autonomy, more than half (54.7%) of them had no financial autonomy. Slightly more than two-fifths (42.1%) of the respondents were able to negotiate safer sex with partners. Though the proportion (30.1%) of respondents who were decision-makers for non-use of contraceptives was substantial, the proportion (28.8%) who reported joint decisions with a partner was equally substantial compared to the proportion (18.5%) whose partners decide their contraceptive non-use. The majority of the respondents were not visited by family planning workers in the last 12 months preceding the survey. Though more than half of the women rejected wife-beating under any circumstance more than two-fifths of them justified wife-beating. The majority of the respondents had never experienced a pregnancy termination. Women from the northern region particularly the northwest geo-political zone were preponderant in the sample.

### Bivariate Results

Table 2 presents the bivariate findings. Health service contacts were significantly associated with the intention to use modern contraceptives with a higher proportion of the intention to use contraceptives among women who had health service contacts with CHWs compared to women who reported otherwise (38.6% vs. 27.2%). The intention to use contraceptives increased from 34.6 to 35.4% as the maternal age group increased from 15 to 24 years to 25–34 years but decline drastically to 19.5% among women in the advanced age group. The uOR at advanced maternal age (uOR = 0.459; 95% CI: 0.406–0.519) confirms a significant negative association between the maternal age group and the intention to use modern contraceptives. Though the intention to use modern contraceptives was lower among women who reported early marriage compared to those who reported otherwise (28.8% vs. 30.0%), the association was however not significant. Parity and the intention to use modern contraceptives were negatively associated. As women’s parity status was increasing from primiparity to multiparity and to grand multiparity, the intention to use modern contraceptives was declining steadily. With the exclusion of women who wanted no more children, the intention to use modern contraceptives was lower (25.3%) among women who wanted another child within the next 2 years compared to women who either wants after 2 years (38.6%), or were undecided (27.5%) or unsure (36.5%) of the timing of future fertility.

**Table 2:** The intention to use contraceptives (%) by demographic and social characteristics and unadjusted odds ratio

Characteristic	Per cent	uOR	95% CI	Characteristic	Per cent	uOR	95% CI
**Health service contacts**				**Religion**			
No contact with CHW ^RC^	27.2	–	–	Christianity ^RC^	35.0	–	–
Contact with CHW	38.6	1.681**	1.440–1.963	Islam	26.8	0.678**	0.531–0.790
**Maternal age group**				Others	26.8	0.677*	0.362–1.267
15–24 years ^RC^	34.6	–		**Household wealth quintile**			
25–34 years	35.4	1.033	0.919–1.159	Poorest ^RC^	25.3	–	–
35+	19.5	0.459**	0.416–0.519	Poorer	29.9	1.262**	1.072–1.485
**Timing of first marriage**				Middle	33.3	1.472**	1.230–1.762
Early ^RC^	28.6	–	–	Richer	33.4	1.478**	1.191–1.835
Not early	30.0	1.073	0.958–1.201	Richest	26.1	1.040	0.762–1.422
**Parity**				**Partners’ education**			
Primiparity ^RC^	32.6	–	–	None ^RC^	24.4	–	–
Multiparity	30.1	0.893	0.794–1.005	Primary	34.2	1.613**	1.392–1.870
Grand multiparity	26.1	0.735**	0.658–0.821	Secondary	39.1	1.992**	1.749–2.269
**Fertility desire**				Higher	34.2	1.616**	1.293–2.024
Wants within 2 years ^RC^	25.3	–	–	**Financial autonomy**			
Wants after 2 years	38.6	1.850**	1.625–2.107	Not autonomous ^RC^	29.2	–	–
Wants unsure of timing	36.5	1.693**	1.359–2.111	Autonomous	28.8	0.980	0.882–1.088
Undecided	27.5	1.116	0.896–1.391	**Healthcare autonomy**			
Want no more	21.1	0.791	0.684–0.915	Not autonomous ^RC^	28.0	–	–
**Maternal education**				Autonomous	31.2	1.195*	1.056–1.353
None ^RC^	25.6	–	–	**Safe sex negotiation**			
Primary	32.8	1.415**	1.216–1.646	Unable to negotiate ^RC^	25.2	–	–
Secondary	40.8	1.200**	1.716–2.330	Able to negotiate	34.2	1.540**	1.339–1.771
Higher	27.3	1.092	0.787–1.516	**Decision-maker for non-use of contraceptives**			
**Working status**				Woman only	28.2	–	–
Unemployed	26.5	–	–	Partner only	24.6	0.830*	0.691–0.997
Employed	30.2	1.203**	1.072–1.349	Joint	28.5	1.017	0.874–1.182
**Mass media exposure**				Others	34.3	1.329**	1.171–1.509
Low	26.4	–	–				
Moderate	31.4	1.271**	1.121–1.442				
High	36.7	1.612**	1.219–2.131				

RC (Reference category), *p < 0.05, **p < 0.01

Maternal education was positively associated with the intention to use modern contraceptives with a consistent increase in the intention to use modern contraceptives as educational attainment improves. However, the level of intention to use contraceptives dropped as the educational level reached higher education. Working status was significant (uOR = 1.203; 95% CI: 1.072–1.349) and positively associated with the intention to use modern contraceptives with higher intention among employed compared to unemployed women (30.3% vs. 26.5%). Likewise, mass media exposure and the intention to use modern contraceptives were positively related to a consistent increase in the level of intention as exposure to mass media improves. Christian women reported a higher intention to use modern contraceptives compared to women in other religions. Except for the richest household wealth group, the intention to use modern contraceptives increase progressively as the household wealth group improves. This reveals a positive association. Also, the intention to use modern contraceptives increase with improvement in partners’ education but the level of intention dropped at higher educational attainment. While healthcare autonomy was positively associated with the intention to use a modern contraceptive, financial autonomy was negatively associated with the intention to use modern contraceptives. Safer sex negotiation relates positively to the intention to use modern contraceptives. In contrast, the association between decision-makers for the non-use of contraceptives and the intention to use contraceptives was inconsistent.

### Multivariable Results

Table 3 presents further association of health service contacts and other characteristics with the intention to use modern contraceptives. In Model 1 which controlled for the individual demographic and social characteristics, the odds of the intention to use modern contraceptives were higher among women who had health service contacts with CHWs compared to the intention among women who had no health service contacts with CHWs (aOR = 1.430; 95% CI: 1.212–1.687). In the model, the timing of the first marriage was the only individual characteristic with no significant effect on the intention to use modern contraceptives. With the introduction of household characteristics into Model 2, two noticeable changes were observed. One, the odds of the intention to use modern contraceptives reduced from 1.430 in Model 1 to 1.358 in Model 2 though the odds remain higher among women who had health service contacts with CHWs compared to those who had no health service contacts (aOR = 1.358; 95% CI: 1.153–1.599). Two, religion no longer reveals a significant effect on the intention to use modern contraceptives. As also observed in Model 1, the timing of the first marriage did not show a significant effect on the intention to use modern contraceptives. Two of the household characteristics, namely, household wealth and healthcare autonomy had no significant effects on the intention to use modern contraceptives. In the full model (Model 3), health service contacts were strengthened by the inclusion of the control variables. Women who had health service contacts with CHWs were more likely to intend to use modern contraceptives compared to women who had no health service contacts with CHWs (aOR = 1.454; 95% CI: 1.240–1.706).

**Table 3:** Effects of health service contacts and other characteristics on the intention to use contraceptives

Characteristic predicting the intention to use contraceptives	Model 1			Model 2			Model 3		
	aOR	***p***-value	95% CI	aOR	***p***-value	95% CI	aOR	***p***-value	95% CI
**Health service contacts**									
No contact with CHW ^RC^	1.000	–	–	1.000	–	–	1.000	–	–
Contact with CHW	1.430**	*p* < 0.001	1.212–1.687	1.358**	*p* < 0.001	1.153–1.599	1.454**	*p* < 0.001	1.240–1.706
**Maternal age group**									
15–24 years ^RC^	1.000	–	–	1.000	–	–	1.000	–	–
25–34 years	0.837*	0.020	0.721–0.973	0.846*	0.027	0.729–0.981	0.902	0.184	0.775–1.050
35+	0.358**	*p* < 0.001	0.294–0.437	0.373**	*p* < 0.001	0.305–0.456	0.399**	*p* < 0.001	0.325–0.491
**Timing of first marriage**									
Early ^RC^	1.000	–	–	1.000	–	–	1.000	–	–
Not early	1.081	0.196	0.960–1.217	1.057	0.351	0.941–1.188	1.145*	0.024	1.018–1.288
**Parity**									
Primiparity ^RC^	1.000	–	–	1.000	–	–	1.000	–	–
Multiparity	1.144	0.062	0.993–1.317	1.146	0.061	0.994–1.323	1.114	0.142	0.964–1.288
Grand multiparity	1.581**	*p* < 0.001	1.321–1.891	1.619**	*p* < 0.001	1.352–1.940	1.497**	*p* < 0.001	1.237–1.765
**Fertility desire**									
Wants within 2 years ^RC^	1.000	–	–	1.000	–	–	1.000	–	–
Wants after 2 years	1.697**	*p* < 0.001	1.492–1.930	1.686**	*p* < 0.001	1.483–1.916	1.790**	*p* < 0.001	1.578–2.031
Wants unsure of timing	1.350	0.009	1.078–1.690	1.396**	0.004	1.111–1.754	1.692**	*p* < 0.001	1.362–2.102
Undecided	1.258	0.065	0.986–1.606	1.233	0.083	0.973–1.563	1.370**	0.006	1.096–1.713
Want no more	0.942	0.543	0.775–1.144	0.933	0.484	0.769–1.133	1.074	0.447	0.894–1.290
**Maternal education**									
None ^RC^	1.000	–	–	1.000	–	–	1.000	–	–
Primary	1.334**	*p* < 0.001	1.110–1.603	1.122	0.197	0.942–1.338	1.256*	0.005	1.071–1.473
Secondary	1.527**	*p* < 0.001	1.234–1.889	1.307*	0.011	1.062–1.608	1.503**	*p* < 0.001	1.239–1.823
Higher	1.029	0.887	0.692–1.529	1.032	0.979	0.685–1.554	1.133	0.524	0.770–1.668
**Working status**									
Unemployed	1.000	–	–	1.000	–	–	1.000	–	–
Employed	1.213**	*p* < 0.001	1.056–1.394	1.379**	0.001	1.138–1.669	1.483**	*p* < 0.001	1.228–1.792
**Mass media exposure**									
Low	1.000	–	–	1.000	–	–	1.000	–	–
Moderate	1.189*	0.013	1.037–1.363	1.166*	0.033	1.012–1.342	1.168*	0.034	1.010–1.352
High	1.412*	0.045	1.008–1.980	1.394	0.058	0.983–1.965	1.540*	0.018	1.076–2.204
**Religion**									
Christianity ^RC^	1.000	–	–	1.000	–	–	1.000	–	–
Islam	0.685*	0.005	0.525–0.894	0.844	0.241	0.635–1.121	0.510**	*p* < 0.001	0.390–0.667
Others	0.654	0.214	0.335–1.279	0.710	0.354	0.344–1.465	0.539*	0.024	0.315–0.920
**Household wealth quintile**									
Poorest ^RC^				1.000	–	–	1.000	–	–
Poorer				1.115	0.194	0.946–1.313	1.100	0.236	0.939–1.289
Middle				1.083	0.407	0.897–1.308	1.191	0.064	0.989–1.434
Richer				0.977	0.855	0.765–1.249	1.195	0.168	0.927–1.540
Richest				0.735	0.066	0.529–1.021	0.973	0.876	0.690–1.372
**Partners’ education**									
None ^RC^				1.000	–	–	1.000	–	–
Primary				1.518**	*p* < 0.001	1.289–1.786	1.525**	*p* < 0.001	1.301–1.787
Secondary				1.527**	*p* < 0.001	1.315–1.773	1.498**	*p* < 0.001	1.287–1.743
Higher				1.462**	0.004	1.131–1.889	1.254	0.078	0.924–1.614
**Financial autonomy**									
Not autonomous ^RC^				1.000	–	–	1.000	–	–
Autonomous				0.794**	0.005	0.675–0.934	0.797**	0.002	0.661–0.912
**Healthcare autonomy**									
Not autonomous ^RC^				1.000	–	–	1.000	–	–
Autonomous				0.980	0.779	0.850–1.129	1.091	0.233	0.945–1.259
**Safe sex negotiation**									
Unable to negotiate ^RC^				1.000	–	–	1.000	–	–
Able to negotiate				1.369**	*p* < 0.001	1.169–1.606	1.434**	*p* < 0.001	1.213–1.696
**Decision-maker for non-use of contraceptives**									
Woman only ^RC^				1.000	–	–	1.000	–	–
Partner only				0.846	0.070	0.705–1.014	0.932	0.429	0.782–1.110
Joint				0.996	0.963	0.851–1.158	1.045	0.553	0.903–1.208
Others				1.288**	0.001	1.114–1.489	1.371**	*p* < 0.001	1.188–1.583
**Visitation by family planning worker**									
Not visited ^RC^							1.000	–	–
Visited							0.971	0.736	0.821–1.150
**Attitude to wife-beating**									
Not acceptable ^RC^							1.000	–	–
Acceptable							1.032	0.653	0.899–1.184
**Pregnancy termination**									
Never experienced ^RC^							1.000	–	–
Ever experienced							0.997	0.974	0.860–1.157
**Geo-political zone of residence**									
North-central ^RC^							1.000	–	–
North-east							1.317*	0.033	1.023–1.625
North-west							2.182**	*p* < 0.001	1.714–2.776
South-east							0.576*	0.003	0.402–0.827
South-south							0.517**	*p* < 0.001	0.387–0.690
South-west							0.604*	0.011	0.410–0.892

RC (Reference category), *p < 0.05, **p < 0.01

In the full model, all the individual demographic and social characteristics revealed a significant association with the intention to use modern contraceptives. While maternal age group and religion reduce the odds of the intention to use modern contraceptives, other individual characteristics, namely, the timing of first marriage, parity, fertility desire, maternal education, working status, and exposure to mass media increases the odds of the intention to use modern contraceptives. On one hand, two household characteristics, namely, household wealth and healthcare autonomy remained without statistical significance as observed in the previous model. On the other hand, other household characteristics, namely, partners’ education, financial autonomy, safer sex negotiation, and decision-maker for non-use of contraceptives reveal significant association with the intention to use modern contraceptives. Geo-political zone of residence was the only control variable that showed significant effects on the intention to use modern contraceptives with higher odds in the North-East and North-West compared to lower odds in the three Southern zones.

## Discussion

This study examined the extent of the association between health service contacts with CHWs and the intention to use modern contraceptives in settings with limited access to skilled health personnel such as doctors and nurses/midwives. This study builds on existing works that have established the important roles of CHWs in boosting contraceptive prevalence [30–32, 36] and was carried out to provide more information on an additional initiative that may boost modern contraceptive usage in rural and remote communities of Nigeria in light of the grossly insufficient numbers of skilled health personnel in such communities [19, 20], which makes the CHWs an important provider of reproductive health services in such communities [13, 23, 24]. This presents an opportunity for family planning programmers to leverage the patronage of CHWs for the purpose of family planning demand generation by encouraging CHWs to use every health service contact with the rural populace to talk about family planning, clear family planning misconceptions, and provide enlightened information about the health concerns of family planning usage. Such initiative will go a long way to reduce the existing disparity not only between the rural and urban areas [1] but also current state and regional disparities [2, 6] in family planning knowledge and use in the country, and also accelerate the achievement of the national contraceptive targets sets for 2030 and 2050 in Nigeria [10].

The study found a 29.0% prevalence of the intention to use modern contraceptives. When compared to findings in existing studies, the prevalence revealed in the current study could be explained in two possible ways. One, the prevalence found is substantially lower than the 52.2, 44.11, and 44.1% respectively reported in three studies conducted outside Nigeria [41, 42, 44] which analyzed similar DHS datasets, and lower than the prevalence found in a Nigerian study [47] that also analyzed the NDHS dataset. It is possible to explain the differences in the prevalence by the group of women analyzed in the studies. While this study focused on rural women who only had health service contacts with CHWs, the other studies conducted outside Nigeria included all childbearing women, and the study conducted in Nigeria included parous women in both urban and rural areas of the country. The prevalence as found in this study may thus be expected to differ from those found in studies that covered larger groups of women. Two, the prevalence found in the current study also differs from the prevalence reported in an existing hospital-based study [43]. Usually, hospital-based studies covered a smaller proportion of childbearing women which often makes the result inconsistent with findings in population-based studies.

Notwithstanding, the prevalence of the intention to use modern contraceptives among non-users as found in this study represents a key opportunity for family planning demand generation in the country. Since CHWs mostly live in the community, they have an understanding of the reproductive norms and culture of the people. This makes them suitable for presenting family planning in a way that is sensitive to the culture, religion, and tradition of the indigenous people, particularly during health service contacts of rural women and may justify higher intention to use modern contraceptives as found among women who had health service contacts with CHWs in the study. The implementation of the family planning blueprint in Nigeria [11, 12] will become more effective by expanding the range of family planning services that CHWs may provide. Incidentally, the existing strategy in the country has empowered CHWs to administer injectable contraceptives [11] but more roles could still be assigned particularly in making resources available for more home visits and family planning outreach programs in rural communities. A recent study in Nigeria [33] confirmed that home visits of CHWs have a strong impact on the uptake of modern contraceptives, which indicates that the strategy is very useful for improving knowledge and the use of modern contraceptives. Though the intention to use modern contraceptives does not automatically translate to future use due to a number of health service factors such as inadequate health services providing contraceptive services, service provision bias, poor private sector participation, and ineffective supply chain management [12], once knowledge is improved, and the intention to use is established, actual use may not face difficult opposition.

Beyond health service contacts with CHWs, this study also found that individual demographic and social characteristics such as age, parity, maternal education, work status, mass media exposure, fertility desire, and religion are important drivers of the intention to use modern contraceptives. This provides support for findings in several existing studies conducted in Nigeria and elsewhere [41, 46, 47]. Also, the study revealed that several relational characteristics such as partners’ education, women’s financial autonomy, safer sex negotiation, and participation in household decisions may have a strong influence on the intention to use modern contraceptives in line with findings in previous studies [42, 43]. The import of these findings is that the socio-demographic context of rural women cannot be exonerated from poor use or non-intention to use modern contraceptives. In most rural communities of Nigeria, the social structure is largely patriarchal which subjugates women’s economic and reproductive lives to male authority. A social transformation of society through improved education and economic empowerment of rural women is a veritable platform for changing the family planning situation in rural and remote communities of Nigeria. This point has been stressed in previous studies [48–51] that call for increasing women’s empowerment to improve the sexual and reproductive health of women in developing countries.

### Strengths and Limitations

The literature search confirms that the focus of this study has rarely been examined across developing countries where CHWs play important role in the provision of primary health care. The study thus expands the frontiers of knowledge about the relevance of CHWs in relation to improvement in modern contraceptive usage in rural communities. The use of the DHS datasets in the study provides a basis for the international comparability of the study methods and findings. The principal investigator will be willing to share the do file with any interested researcher to enhance its replication in other climes. It is also important to draw attention to the few drawbacks of the study. One, the health service contacts recognized in the analysis were limited to contacts for maternal healthcare services such as antenatal care, delivery, postnatal check for mothers, or postnatal check for a child. Health services contacts for other health issues were excluded. This was done to ensure that the women concerned are those susceptible to pregnancy and child delivery concerns which makes family planning a relevant matter to them. The inclusion of other health contacts may moderate the findings. Also, the data analyzed did not capture the possibility of some women contacting both doctors/nurses and CHWs, which may further moderate the findings. In addition, it is important to note that the study did not exclude women based on the recency of last delivery. This is important because the intention to use contraceptives as measured in the study focused on the likely future use of contraceptives and not necessarily the intention to use contraceptives after the last delivery.

Two, the cross-sectional nature of the analyzed data did not permit the establishment of cause and effect between health service contacts and the intention to use modern contraceptives. It, however, provides a significant association between them which is important for understanding how health service contacts relate to the intention to use modern contraceptives. Three, the application of the theory of planned behavior may have improved the study findings, however, the theory could not be applied due to the unavailability of variables that could appropriately measure most of the theoretical constructs. Finally, the study only considered a binary measure of health service contacts. The number of contacts was not captured in the analyses. Follow up studies may thus include the number of health service contacts as another exposure variable that may affect the intention to use contraceptives.

## Conclusion

This study examined whether health service contacts with CHWs are significantly associated with the intention to use modern contraceptives among non-users in rural communities of Nigeria. The women’s datasets of the most recent Nigeria Demographic and Health Survey were analyzed based on a sample of 12,140 rural women. Findings from the analysis revealed that more than a quarter of the rural women intend to use modern contraceptives, and the odds of intention to use modern contraceptives were higher among women who had health service contacts with CHWs. In rural areas of Nigeria, health service contacts with CHWs are significantly associated with the intention to use modern contraceptives. This implies that family planning programmers should leverage the patronage of CHWs for the purpose of family planning demand generation in rural areas of Nigeria.

## Data Availability

Interested researchers and members of the public could access the dataset online at. https://dhsprogram.com/data/dataset/Nigeria_Standard-DHS_2018.cfm?flag=1.

## Abbreviations

AOR: Adjusted Odds Ratio
CHWs: Community Health Workers
DHS: Demographic and Health Survey
FMoH: Federal Ministry of Health
NDHS: Nigeria Demographic and Health Survey
NPC: National Population Commission
